# Reliability of different smartphones measuring the hallux valgus parameters in a new rapid method: a follow-up study

**DOI:** 10.1186/s12891-022-05217-9

**Published:** 2022-04-02

**Authors:** Lin Wang, Chao Zhang, Hao Liang, Jun Zhang, Weiyang Zhong, Zenghui Zhao, Tianji Huang, Xiaoji Luo

**Affiliations:** 1 Department of Orthopedic Surgery, The First Affiliated Hospital of Chongqing Medical University, Chongqing, 400016 People’s Republic of China; 2 Orthopedic Laboratory of Chongqing Medical University, Chongqing, 400016 People’s Republic of China

**Keywords:** Hallux valgus, Smartphones, Measurement, HVA, IMA

## Abstract

**Objective:**

This study aimed to further compare the abilities to measure hallux valgus parameters in different smartphones using the intrinsic photograph-editing function.

**Methods:**

We retrospectively reviewed 61 patients (100 feet) of hallux valgus without medical or surgical interventions at our department. The radiographic parameters were assessed and measured via the Picture archiving and communication systems (PACS), iPhone, and Android. The accuracy, reliability, and the time-taken were compared and analyzed between each two methods.

**Results:**

The mean value of measured hallux valgus parameters were as follow: hallux valgus angle (HVA): 33.71 ± 7.25°; the first and second intermetatarsal angle (IMA): 12.84 ± 3.62° in PACS; HVA: 33.59 ± 7.18° and IMA: 12.80 ± 3.65° in Android; HVA: 33.63 ± 7.23° and IMA: 12.87 ± 3.60° in iPhone. No significant difference was found among the average results measured by PACS, Android and iPhone (*F* = 0.008, *P* = 0.992 in HVA; *F* = 0.009, *P* = 0.991 in IMA). For measurements by PACS, Android smartphone, and iPhone, the variability of HVA (*F* = 0.061, *P* = 1.000) and IMA (*F* = 0.133, *P* = 1.000) was similar. The intraclass correlation coefficients (ICCs) of the mean results of four times measurements of HVA and IMA as follows: PACS vs Android: 0.995 (0.993–0.997) and 0.982 (0.973–0.988); PACS vs iPhone:0.997 (0.995–0.998) and 0.974 (0.962–0.982); Android vs iPhone:0.997 (0.995–0.998) and 0.981 (0.971–0.987). The interobserver and intraobserver reliability was very good for Android smartphones and iPhone in measuring hallux valgus parameters. The mean time of measurement by PACS, Android smartphone, and iPhone were 25.34 ± 1.18 s, 20.10 ± 0.92 s, and 19.92 ± 0.99 s respectively. The measurement time of smartphones is significantly faster than that of PACS by about 5 seconds (*P* = 0.000). The measurement time of iPhone was slightly faster than that of Android smartphone, while no significant difference was found (*P* = 0.24).

**Conclusion:**

It is more convenient and faster to use smartphones when compared with PACS, at the same level of accuracy. Furthermore, the abilities of different smartphone platforms are proven to be of no significant difference.

## Introduction

Hallux valgus (HV) is one of the most commonly seen complex deformities of the foot in clinical and is featured as abnormal angulations, deviations, and rotations of the big toe, which always lead to significant functional disability and foot pain [1]. Although it may easily be identified and diagnosed in clinical practice, the etiology of HV remains elusive. According to previous accumulating studies, the etiologies could be usually categorized as extrinsic (e.g., high-heels and narrow-toe-box shoes) and intrinsic (e.g., long first metatarsal, the shape of the metatarsal head) causes [2]. This may be the main underlying reason why the multitude of surgical procedures existed in clinical decisions [2, 3]. It was reported that the pooled prevalences of HV were 23% for adults aged 18–65 years and 35.7% for the elderly. It also showed a predilection towards women, resulting from differences in lifestyles [1]. The radiographic parameters of HV are considered as one of the most crucial clinical tools to diagnose, classify the severity of the deformity, select the appropriate surgical treatment and evaluate the postoperative outcome [4]. The most commonly used radiographic parameters in assessing patients with HV are the hallux valgus angle (HVA), the first and second intermetatarsal angle (IMA), the distal metatarsal articular angle (DMAA) [5]. Among them, the most important and commonly used angles for assessing the severity of HV are HVA (normal:<15°; mild: <20°; moderate: 20–40°; severe: >40°) and IMA (normal:<9°; mild: 9–11°; moderate: 12–16°; severe: >16°), which were validated to be of good reliability and reproducibility [3, 5, 6]. Conventionally, the protractor was used to measure the included angle between each two labeled lines which were drawn on the radiograph. However, the conventional way has been proved to be error-prone and time-consuming [7–9]. In the past few years, computer-assisted image analysis software and smartphone applications have become widespread in daily use, in which the Picture archiving and communication systems (PACS) were considered as the gold standard. Some applications of iPhone have been developed and described to measure the angles of hallux valgus in previous researches [10–13]. In our previous study, we introduced a brand-new method of measuring the hallux valgus angles conveniently and rapidly with the intrinsic photograph-editing function of Android smartphones [14]. However, there were no relevant clinical studies to further explore the discrepancies of accuracy and reliability in measuring radiographic parameters via different intelligent devices. We hypothesized that different smartphones share the same accuracy of measuring angles, compared with PACS. Beyond that, the time required for measuring in smart devices was far less than PACS. Therein, iPhone may spend a shorter time-scale than the Android smartphone due to a better graphics processor. The aim of this study was to test this hypothesis.

So in this study, we applied different smartphones which mainly include Android smartphones and iPhone to assess and measure the radiographic parameters of hallux valgus. Moreover, the accuracy, repeatability and measuring time were assessed and compared.

## Material and methods

### Selection of patients

We retrospectively reviewed 61 patients of hallux valgus without medical or surgical interventions at our department, and extracted the anteroposterior weight-bearing radiographs from PACS. This research is a follow-up study to our previous study, which was approved by the ethics committee of The First Affiliated Hospital of Chongqing Medical University. Informed consent was obtained from all patients.

### Methods

**Fig. 1:**
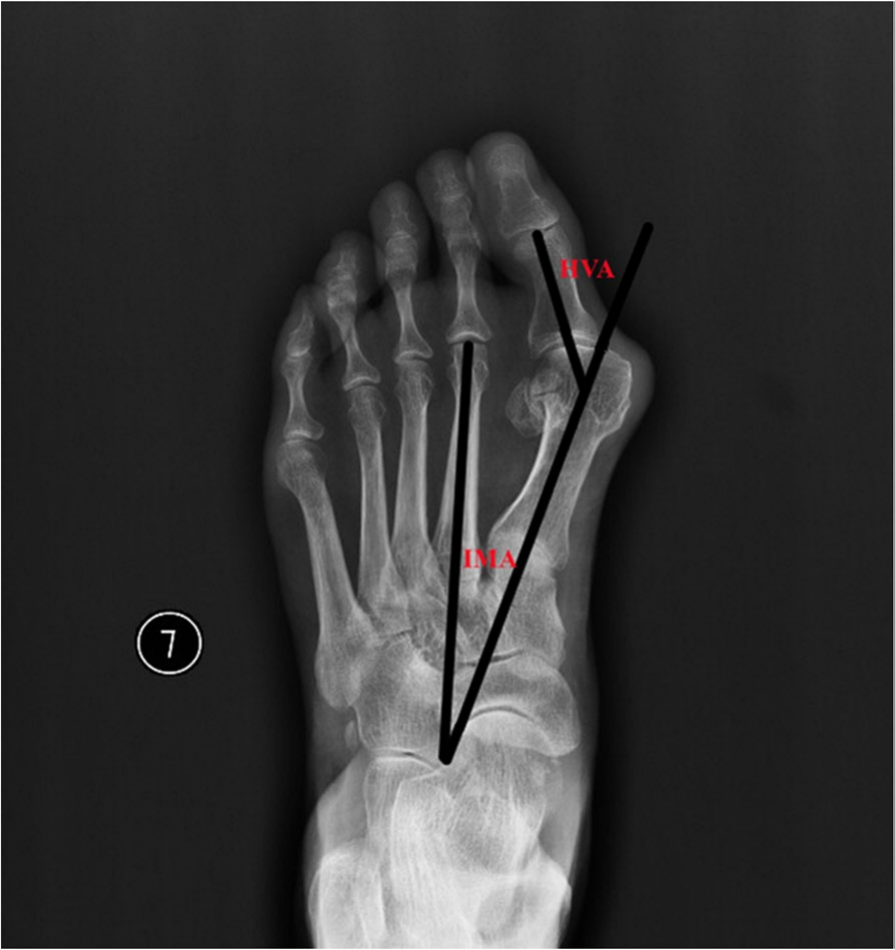
Angular measurements of HVA and IMA

The build-in image processing tools of PACS were used to measure the included angles. All the measurements were carried out according to the ad hoc committee of the American Orthopaedic Foot and Ankle Society (Fig. 1) [15]. Furthermore, the measuring outcomes were defined as the reference value to our statistical analysis and comparison.

**Fig. 2:**
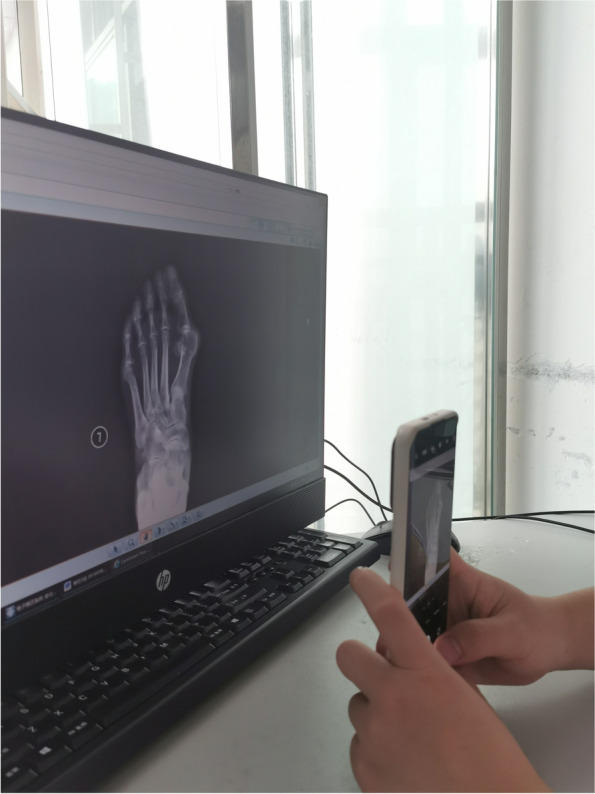
The camera must be parallel to the monitor strictly during the procedure to avoid parallax errors

**Fig. 3:**
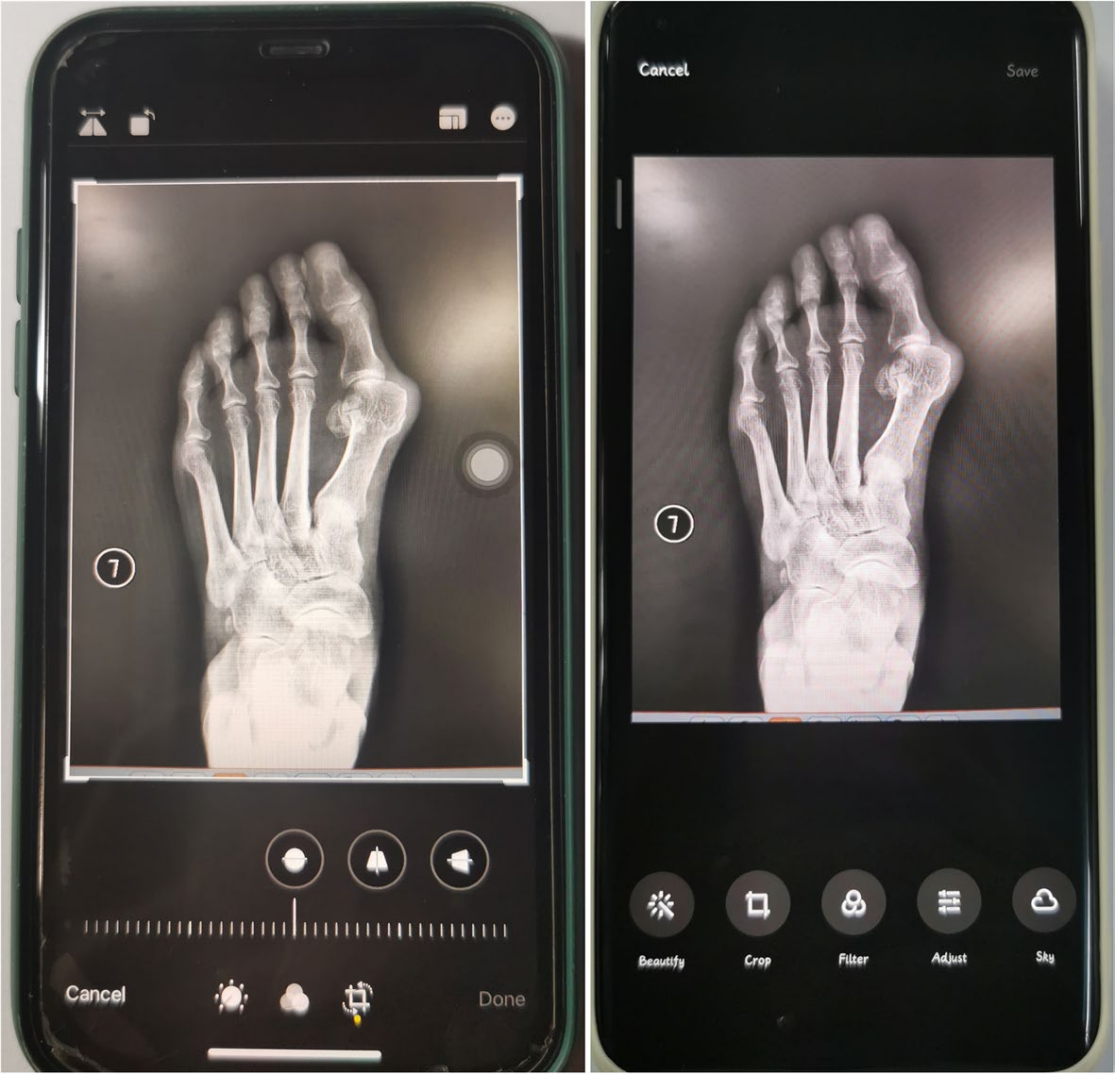
The intrinsic photograph-editing function of different smartphones (the left one represented Android, the other was iPhone)

As for the smartphone measuring, we adopted the procedure used in our previous study [14]. Firstly, as Fig. 2 shows, we take pictures without any markers from the PACS system, and the camera must be parallel to the monitor strictly during the procedure. Secondly, observers rotate the image until the grid lines could be seen perfectly using the intrinsic photograph-editing function (Fig. 3).

**Fig. 4:**
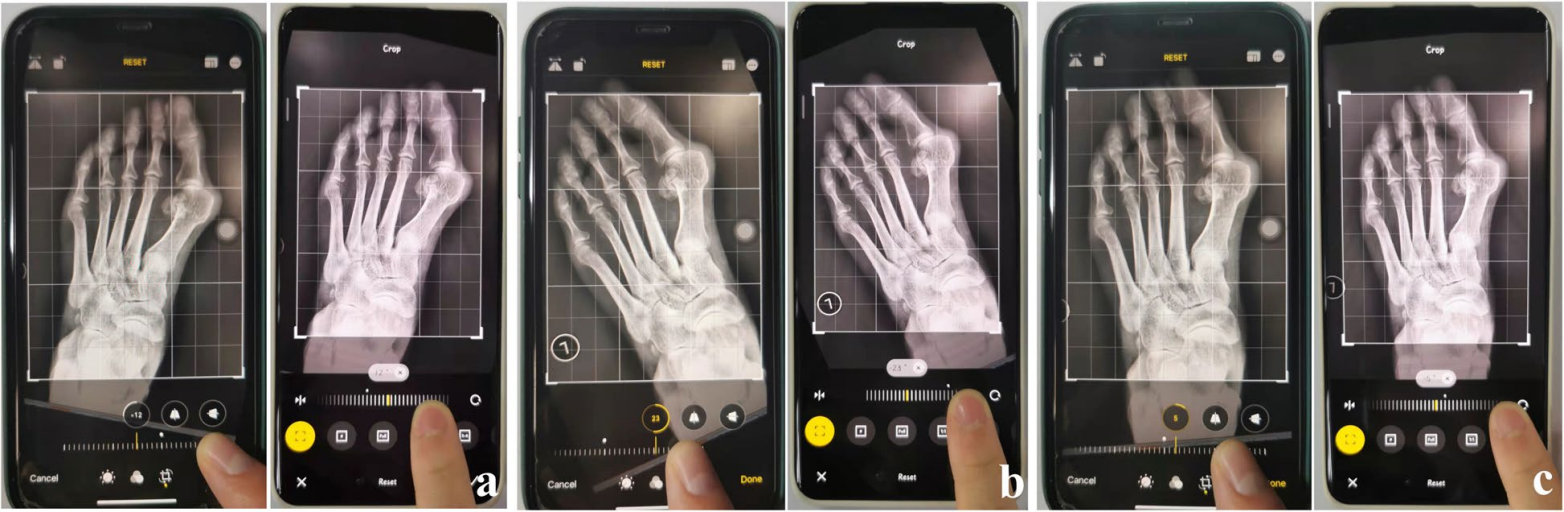
The methods of measuring HVA and IMA. **a** Rotate the photo until the gridline was parallel or overlapping to the long axis of the first proximal phalanx. **b** Get the gridline parallel or overlapping to the long axis of the first metatarsal bone. **c** Rotate the photo to get gridline parallel or overlapping to the second metatarsal bone

With measuring HVA as an example, firstly, observers rotated the photo until the gridline was parallel or overlapping to the long axis of the first proximal phalanx (Fig. 4a); then, this rotation angle was recorded as Angle1. Secondly, get the gridline parallel or overlapping to the long axis of the first metatarsal bone in the same way (Fig. 4b); then, the rotation angle was recorded as Angle2. The HVA is defined as the difference between these two angles.

The method of measuring IMA is similar. The rotation angle was named Angle3 when the gridline was parallel or overlapping to the second metatarsal bone (Fig. 4c). The difference between Angle2 and 3 was registered as IMA.

All the measurements were carried out by two experienced orthopaedists from our department independently. The time measurements were recorded with a stopwatch.

The measurement sequence of the two observers is shown below: firstly, observers used PACS to measure the HVA and IMA in Week 1, the results were recorded as PACS A t1 and PACS B t1; then measuring the HVA and IMA by Android in Week2, the results were recorded as Android A t1 and Android B t1; then the iPhone was used by two observers to measure in Week 3; the results were recorded as iPhone A t1 and iPhone B t1.

Finally, the measurements were repeated during the following 3 weeks in the order described above and were recorded as t2.

In order to minimize the possible recall and deviation, measurements were presented in randomized order during each trial. Moreover, all the measurements were kept by the third party. A total of 1200 HVAs and 1200 IMAs (100 ft were measured by two observers with three methods twice) were recorded and the average value was calculated in Excel 2016.

### Analysis

All statistical analyses were performed in a blinded manner. All the data were analyzed by SPSS 21.0. Kolmogorov-Smirnov test was used to check whether the values were normally distributed. One-way ANOVA was used to compare the difference among every measurement by different observers and different methods. The time difference of different measurement methods was also compared by One-way ANOVA. The reliability of the three kinds of measurement methods was analyzed by two-way random intraclass correlation coefficients (ICCs). ICC values divided reliability into five levels: poor reliability (0.00–0.20), fair reliability (0.21–0.40), moderate reliability (0.41–0.60), substantial or good reliability (0.61–0.80) and very good reliability (0.81–1.00) [10].

## Results

This study reviewed radiographs of 100 feet (53 left and 47 right) from 61 patients (11 males and 50 females). The average age of all the patients was 58.7 ± 11.8 years old. The average result of HVA measured by PACS was 33.71 ± 7.25°, and the average result of IMA measured by PACS was 12.84 ± 3.62°. The average result of HVA measured by Android smartphone was 33.59 ± 7.18°, and the average result of IMA measured by Android smartphone was 12.80 ± 3.65°. The average result of HVA measured by iPhone was 33.63 ± 7.23°, and the average result of IMA measured by iPhone was 12.87 ± 3.60°. No significant difference was found among the average results measured by PACS, Android smartphone and iPhone (HVA: *F* = 0.008, *P* = 0.992; IMA: *F* = 0.009, *P* = 0.991), indicating that both Android smartphone and iPhone could also be applied to measure the hallux valgus parameters. For measurements by PACS, Android smartphone and iPhone, the variability was similar (HVA:*F* = 0.061, *P* = 1.000; IMA:*F* = 0.133, *P* = 1.000) (Table 1). The ICCs of the mean results of four times measurements of HVA and IMA between PACS measurement and Android smartphone measurement were 0.995 (0.993–0.997) and 0.982 (0.973–0.988), the ICCs of the mean results of four times measurements of HVA and IMA between PACS measurement and iPhone measurement were 0.997 (0.995–0.998) and 0.974 (0.962–0.982), the ICCs of the mean results of four times measurements of HVA and IMA between Android smartphone measurement and iPhone measurement were 0.997 (0.995–0.998) and 0.981 (0.971–0.987), demonstrating that the three kinds of measurement (PACS, Android smartphone and iPhone) were highly correlated. Tables 2, 3 and 4 shows the ICCs results of every measurement by PACS, Android smartphone and iPhone, revealing that the concordance among these three kinds of measurement were very good for measuring parameters in hallux valgus radiographs. The ICCs results in the Tables 5 and 6 indicated that the interobserver and intraobserver reliability was very good for both Android smartphone and iPhone when measuring HVA and IMA in hallux valgus radiographs. The mean time of measurement by PACS, Android smartphone and iPhone were 25.34 ± 1.18 s, 20.10 ± 0.92 s and 19.92 ± 0.99 s respectively. The results of One-way ANOVA indicated that both the measurement time of Android smartphone and iPhone were significantly faster than the measurement time of PACS by about five seconds (*P* = 0.000). The measurement time of iPhone was slightly faster than that of Android smartphone while no significant difference was found (*P* = 0.24).

**Table 1:** Comparison of measurements by PACS, Android smartphone and iPhone

	HVA (°)	IMA (°)
PACS A t1	33.42 ± 7.13	12.68 ± 3.64
PACS A t2	33.49 ± 7.10	12.82 ± 3.82
PACS B t1	33.56 ± 7.28	12.77 ± 3.70
PACS B t2	33.55 ± 7.24	12.78 ± 3.77
Android A t1	33.74 ± 7.39	12.79 ± 3.84
Android A t2	33.70 ± 7.21	13.16 ± 4.13
Android B t1	33.96 ± 7.44	12.97 ± 3.60
Android B t2	33.40 ± 7.21	12.75 ± 3.66
iPhone A t1	33.70 ± 7.33	12.67 ± 3.55
iPhone A t2	33.92 ± 7.38	12.91 ± 3.78
iPhone B t1	33.62 ± 7.23	12.76 ± 3.68
iPhone B t2	33.52 ± 7.22	12.83 ± 3.56
Significance (*P* value)	1.000	1.000

PACS B: the results of observer B using PACS

iPhone A: the results of observer A using iPhone

t1: the first time of observation

t2: the second time of observation

**Table 2:** Comparison of every measurement indicated the reliability of the results by PACS and Android smartphone is very good

	Observer A t1		Observer A t2		Observer B t1		Observer B t2	
	ICC (95% CI)	*P*	ICC (95% CI)	*P*	ICC (95% CI)	*P*	ICC (95% CI)	*P*
HVA	0.983 (0.975–0.989)	0.000	0.982 (0.973–0.988)	0.000	0.982 (0.967–0.990)	0.000	0.986 (0.978–0.991)	0.000
IMA	0.930 (0.897–0.952)	0.000	0.942 (0.915–0.961)	0.000	0.948 (0.923–0.965)	0.000	0.940 (0.913–0.959)	0.000

**Table 3:** Comparison of every measurement indicated the reliability of the results by PACS and iPhone is very good

	Observer A t1		Observer A t2		Observer B t1		Observer B t2	
	ICC (95% CI)	*P*	ICC (95% CI)	*P*	ICC (95% CI)	*P*	ICC (95% CI)	*P*
HVA	0.987 (0.980–0.991)	0.000	0.988 (0.983–0.992)	0.000	0.989 (0.984–0.993)	0.000	0.983 (0.973–0.989)	0.000
IMA	0.947 (0.922–0.964)	0.000	0.812 (0.733–0.870)	0.000	0.967 (0.946–0.979)	0.000	0.945 (0.919–0.963)	0.000

**Table 4:** Comparison of every measurement indicated the reliability of the results by Android smartphone and iPhone is very good

	Observer A t1		Observer A t2		Observer B t1		Observer B t2	
	ICC (95% CI)	*P*	ICC (95% CI)	*P*	ICC (95% CI)	*P*	ICC (95% CI)	*P*
HVA	0.986 (0.980–0.991)	0.000	0.982 (0.973–0.988)	0.000	0.985 (0.977–0.990)	0.000	0.991 (0.987–0.994)	0.000
IMA	0.932 (0.901–0.954)	0.000	0.839 (0.770–0.889)	0.000	0.942 (0.915–0.961)	0.000	0.960 (0.941–0.973)	0.000

**Table 5:** Two observers’ measurements indicated the inter- and intraobserver reliability by android smartphone are very good

	Observer A t1 versus Observer B t1		Observer A t2 versus Observer B t2		Observer A t1 versus Observer A t2		Observer B t1 versus Observer B t2	
	ICC (95% CI)	*P*	ICC (95% CI)	*P*	ICC (95% CI)	*P*	ICC (95% CI)	*P*
HVA	0.974 (0.962–0.983)	0.000	0.984 (0.976–0.989)	0.000	0.974 (0.961–0.982)	0.000	0.989 (0.984–0.993)	0.000
IMA	0.894 (0.847–0.928)	0.000	0.942 (0.916–0.961)	0.000	0.929 (0.897–0.952)	0.000	0.954 (0.932–0.968)	0.000

**Table 6:** Two observers’ measurements indicated the inter- and intraobserver reliability by iPhone are very good

	Observer A t1 versus Observer B t1		Observer A t2 versus Observer B t2		Observer A t1 versus Observer A t2		Observer B t1 versus Observer B t2	
	ICC (95% CI)	*P*	ICC (95% CI)	*P*	ICC (95% CI)	*P*	ICC (95% CI)	*P*
HVA	0.992 (0.988–0.995)	0.000	0.992 (0.988–0.995)	0.000	0.990 (0.985–0.993)	0.000	0.991 (0.987–0.994)	0.000
IMA	0.955 (0.934–0.969)	0.000	0.856 (0.793–0.901)	0.000	0.827 (0.753–0.880)	0.000	0.968 (0.953–0.979)	0.000

## Discussion

Hallux valgus is a complex deformity of the foot that has multiple etiologic factors. As reported, there are more than 100 different operative procedures have been created to treat hallux valgus [2]. So, it is of vital importance to choose the proper treatment modality, which is highly based on clinical assessment and radiographic measurements to define the severity of the deformity. To our knowledge, there are many radiographic parameters that can be used to evaluate the deformity included HVA, IMA, DMAA, MASA, IPA, and MAA. According to the previous studies, the most frequently used imaging parameters in clinical practice are HVA, IMA, and DMAA, in which DMAA was proved to be the less reliable and poor determination of congruency [6, 16]. So we choose HVA and IMA on behalf of other hallux valgus parameters (DMAA, MASA, IPA, and MAA) to complete our research.

Conventionally, measurements were done manually with the assistance of a marker and angle-measuring instrument, which is both cumbersome and prone to human operator error [7–9]. Computer-assisted image analysis software and smartphone applications have become widespread in many areas with the development of science and technology, in which PACS and other familiar systems were highly adapted in medical institutions for their convenience and accuracy. However, not every hospital can afford such an expensive information system in developing countries. Meanwhile, a wide variety of systems added complexity to communication and operation. So, manual measurements are still quite common in our outpatient department. Nowadays, high-tech smartphones have been proven to be a convenient and reliable way to provide accurate digital imaging processing and measuring. As a convenient tool, the smartphone has plenty of innate advantages. The radiograph could be measured and analyzed almost anytime under some special clinical condition, for instance, during the surgery. The surgeon could estimate the effect of surgery and guide the further treatment on time with the assistance of a smartphone. Besides that, a smartphone could also provide a communication platform where doctors can have remote consultation and discussion about illness, and patients can upload their medical records to get a better diagnosis even in the remote area.

As for the measuring HVAs, some iPhone applications had been introduced in previous researches. The Hallux Angles software had been proven to be an accurate and reproducible method compared with the conventional PACS system [11]. Yang et al. found that the Tiltmeter software in iPhone is superior to PACS in measuring time but with the same accuracy [13]. Mattos et al. even showed that one iPhone app named iPinPoint could even be operated by non-experienced observers, which means patients may be able to self-screening such deformities [10]. However, some limits merit further attention: firstly, the software mentioned above are lacks updates. Actually, we found that The Hallux Angles software could not be downloaded now, and iPinPoint had not been updated for nearly a year; secondly, most apps are designed for iPhone and are not free. So, in our previous study, we developed a brand-new measuring method of hallux valgus parameters using the intrinsic photograph-editing function of Android, which is proven to be of accuracy, reliability, and convenience [14]. Nevertheless, we do notice that some researches have showed that iPhone is more accurate than Android phones in orthopedics use for clinical measuring. This aspect remains still open for discussion and research.

In this study, in-depth analysis has been conducted on the difference of results of radiology angle of hallux valgus by using iPhone, Android, and PACS. Consistent with our previous measuring method, the difference of angle parameter and time between every two methods were compared through different types of statistical methods.

Surprisingly, we found that the both interobserver and intraobserver reliability for iPhone and Android were very good, indicating that using the intrinsic photograph-editing function of smartphones is reliable and precise. The analysis of the time required for the measurements indicated that both iPhone and Android are apparently faster than PACS system, which would be a significant advantage over the conventional way. This finding also corroborates our previous research. Besides, we noticed that the measurement time of iPhone was slightly faster than that of Android smartphone while no significant difference was found, which may benefit from the better sensors and smoother operation experience.

Given our present finding, we can conclude that using the intrinsic function of editing photos of smartphones to measure is more convenient and less time consuming. In addition, different smartphone platforms showed surprisingly stability and consistency. These findings merit attention from a public health perspective. Statistically, the Android platform has much larger market share than iPhone, and this gap will maybe even more pronounced in poor and remote areas [17]. So, the same measuring ability of the two different platform is beneficial for the application and spreading in clinical use.

## Conclusion

This study assessed the capabilities of different smartphones using the intrinsic function of photo-editing to measure hallux valgus angles. It is more convenient and faster to use smartphones when compared with PACS, at the same level of accuracy. Furthermore, the abilities of different smartphone platforms are proven to be of no significant difference.

## Data Availability

The datasets generated and/or analyzed during the current study are not publicly available due to the need for further research but are available from the corresponding author on reasonable request.
